# C-954-07. Impact of Preexisting Sleep Disorders on Skin Graft Outcomes and Scarring After Burns

**DOI:** 10.1093/jbcr/irag033.139

**Published:** 2026-04-06

**Authors:** Philong Nguyen, Joshua Wang, Yousef Tanas, Kamryn K Baker, Kevin Vargas, Sudhanvan Iyer, Noelle Gonzalez, Carolyn Henein, Jong Lee

**Affiliations:** University of Texas Medical Branch, Galveston, TX; University of Texas Medical Branch, Galveston, TX; Houston Methodist Hospital, Houston, TX; McGovern Medical School at University of Texas Health Science Center at Houston, Houston, TX; Baylor College of Medicine, Houston, TX; University of Texas Medical Branch, Galveston, TX; University of Texas Medical Branch - John Sealy School of Medicine, Galveston, TX; University of Texas Medical Branch - John Sealy School of Medicine, Galveston, TX; Division of Burn and Trauma Surgery, Department of Surgery, University of Texas Medical Branch, Galveston, TX

## Abstract

**Introduction:**

Sleep disorders have been linked to impaired wound healing and heightened inflammatory responses, but their effect on outcomes after split-thickness skin grafting in burn patients remains unclear. To date, no known study has specifically examined this relationship. This study evaluates the association between preexisting sleep disorders and graft-related complications and scarring using a large national database.

**Methods:**

The TriNetX Research Network was queried to identify patients ages 18 years and older who sustained burn injuries and underwent split-thickness skin grafting between 2010 and 2024. Patients with a documented sleep disorder within one year prior to grafting were matched 1:1 to controls without sleep disorders using propensity scores accounting for demographics, comorbidities, substance use history, psychiatric history, total burn surface area, and grafting procedure. Outcomes included graft failure, infection, and unspecified graft complications. Secondary outcomes included hypertrophic scarring and fibrotic skin conditions. Cumulative incidence, hazard ratios (HR), 95% confidence intervals (CI), and log-rank *p*-values were calculated at 1 and 2 months. Statistical significance was defined as *p*<.05.

**Results:**

After matching, each cohort included 2568 patients. At 1 month, patients with sleep disorders had higher risks of graft failure (3.13% vs 1.07%, HR 3.00, 95% CI 1.91–4.73, *p*<.001), graft infection (0.99% vs 0.41%, HR 2.43, 1.16–5.07, *p*=.015), and unspecified graft complications (2.37% vs 1.37%, HR 1.76, 1.14–2.70, *p*=.009). They also demonstrated increased hypertrophic scarring (7.96% vs 4.53%, HR 1.82, 1.44–2.32, *p*<.001) and fibrotic skin conditions (8.19% vs 5.88%, HR 1.43, 1.14–1.78, *p*=.002). At 2 months, these associations persisted: graft failure (4.32% vs 2.08%, HR 2.16, 1.52–3.05, *p*<.001), graft infection (1.16% vs 0.59%, HR 2.01, 1.06–3.82, *p*=.029), unspecified complications (3.79% vs 2.08%, HR 1.84, 1.30–2.60, *p*=.001), hypertrophic scarring (12.98% vs 8.90%, HR 1.53, 1.28–1.83, *p*<.001), and fibrotic skin conditions (14.45% vs 11.82%, HR 1.26, 1.05–1.51, *p*=.015).

**Conclusions:**

Preexisting sleep disorders are associated with significantly higher risks of graft failure, infection, and hypertrophic scarring following split-thickness skin grafting in burn patients. Prospective studies are needed to confirm causality and clarify underlying mechanisms.

**Applicability of Research to Practice:**

These findings emphasize the importance of sleep health in surgical recovery. Routine screening and perioperative optimization of sleep disorders through multidisciplinary care may reduce complications and improve graft outcomes.

**Funding for the study:**

This study was supported by foundation funding through the Institute for Translational Sciences (UL1 TR001439), funded by the National Center for Advancing Translational Sciences at the NIH.

